# Puerperal Eclampsia

**Published:** 1904-11-19

**Authors:** Robert Jardine

**Affiliations:** Professor of Midwifery in St. Mungo's College, Glasgow, Senior Physician to the Glasgow Maternity Hospital, etc.


### Hospital Clinics.

### PUERPERAL ECLAMPSIA.

By Robert Jardine, M.D., F.R.S.E., Professor of Midwifery in St. Mungo's College, Glasgow, Senior Physician to the Glasgow Maternity Hospital, etc.

In my lecture on albuminuria I mentioned that a patient suffering from bad albuminuria was very liable to be seized with eclampsia. The two conditions are very closely associated. Cases of eclampsia have been reported without any albumen in the urine, but I have never met with one.

The exact cause of the convulsions is unknown. There are many theories, but none of them give a satisfactory explanation. At present all that we can say is that the fits are caused by a toxin or poison which has arisen in connection with the pregnancy, and has gradually accumulated in the system until the nervous system is so affected that convulsions result. In connection with the growth of the uterus and its contents there must be a very large amount of waste products to be got rid of through the natural channels of excretion. What these waste products are we do not know, but we do know that they are poisonous, and that if they are not got rid of they will inevitably kill the patient. The woman's excretory organs have to do double work, so to speak, and if any of them fail a dangerous condition quickly arises. The appearance of albumen in the urine indicates that the kidneys are at fault, but other organs are also affected. The liver is, but the *rôle* it plays is not understood. The kidney condition is the one to which most attention has been paid. Now you may ask is the albuminuria the cause of the poisoning of the system or the result of it? I think there can be little doubt that it is the result of the action of the poison on the kidney substance. The poison acts on the kidney very much like turpentine does. The kidneys are, as you know, very important organs of excretion, and as soon as they begin to fail to do their work properly poisonous waste products rapidly accumulate in the system. In the great majority of cases, the kidneys are not actually diseased, but the condition known as "kidney of pregnancy" is present.

The convulsions may commence during pregnancy, labour, or the puerperium. When they occur during pregnancy, it is usually in the later months; but they are occasionally seen in the early months. I have never seen a case earlier than mid-term, and that was in a case where there was kidney disease present. Primiparæ are much more susceptible to attacks than multiparæ. Elderly multiparæ seem more liable to attacks than those under forty. Thus, it is towards the beginning and towards the end of the child-bearing period that this disease shows itself most frequently.

The patient usually shows dropsy of the face, body, legs, feet, and sometimes of the vulva. She is dull, has severe headaches, her vision is affected, sometimes to the extent of total blindness. She often complains of pain in the stomach, with or without sickness. The urine will be scanty or even suppressed, and it will be loaded with albumen, and, not unfrequently, contains a large amount of blood.

When the fits occur, they exactly resemble epileptic ones, but the patient rarely cries out, and has no warning of the onset, whereas an epileptic often has what is known as an "aura," or warning. She is very likely to bite her tongue, and, if she does so, blood-stained froth will issue from her mouth. She struggles violently for a minute or two, and then lies, for a time, in a more or less comatose condition. She may have only one fit, but as a rule it is repeated, and she may have a large number. Coma usually deepens as the fits recur. After the fits have ceased, the patient may become conscious for a short time; but, as a rule, the coma lasts for some hours, and, in bad cases, I have known it last for three days. When consciousness returns, the patient has no recollection of anything that has occurred to her since the onset of the fits.

### Diagnosis.

If fits occur during pregnancy, labour, or the puerperium in a patient who is not an epileptic, and, at the same time, there is albumen in the urine, you may be sure the case is one of eclampsia. Hysterical fits may simulate eclampsia, but the patient will take good care not to bite her tongue and she will generally be noisy.

### Prognosis.

The condition is a very serious one. The risk is greatest in cases occurring during pregnancy and least in those occurring during the puerperium. The most serious cases are not always those with a large number of fits, neither are they those with the largest amount of albumen in the urine. If little or no urine is being passed and the kidneys cannot be got to act promptly, the case will be a hopeless one. The prognosis for the child is also very grave. It may die in utero, during delivery, or shortly after it is born. Its urine will often contain albumen. Roughly speaking one may say that 20 per cent. of the mothers and 50 per cent. of the children are lost.

As a rule attacks do not recur in future pregnancies, but there are exceptions in about 1 per cent. of cases. In recurrent cases the probability is the kidneys are diseased.

### Treatment.

The following treatment is the one I have used for some years. It has given me better results than any other. It is based on the belief that the fits are caused by the action of a poison on the system. If we knew the poison we could probably find an antidote, but until its nature is discovered the most rational method of treatment is to get rid of the poison through the natural channels of excretion. These channels are the bowels, the kidneys, and the skin, and if we can get all of these to act well the patient will be saved.

In the treatment of the patient the first point to be attended to is to prevent her from biting her tongue or injuring herself in her struggles. A cork, piece of india-rubber, a folded towel, etc., may be placed between her teeth, and the struggles should be so far restrained as to prevent injury, but great force must not be employed. Of course, if the spot where the patient has fallen is a dangerous one, she must be removed from it. It is well to bear in mind that an injury may happen when she falls. Last summer I saw a patient in consultation. After she was delivered I found that she had dislocated her right shoulder. When seized she was in the bathroom, and had fallen against the bath.

It is very important to get the bowels moved freely. If the patient can swallow, she should be given a large dose of opening medicine. I give from two to three heaped tablespoonfuls of Epsom salts. When the patient is comatose, the dose should be given through a stomach-tube. Croton-oil is often used, but it is very uncertain in action, and it may cause œdema of the glottis. The lower bowel should be cleared by means of a copious enema of soap and water. To get the kidneys to act I give large saline injections, usually under the breast. Each injection consists of two or three pints of the following solution : 1 drachm of acetate of soda and 1 drachm of chloride of soda to each pint. The solution is quickly absorbed, and, while it dilutes the poison and flushes the kidneys, it also stimulates the patient. If the patient can swallow, she should be given large quantities of Imperial drink.

To act upon the skin, a hot bath or hot pack should be employed. The hot pack has a wonderfully soothing effect, and a very restless patient will often lie quite quiet in the pack. In giving a hot pack, be sure that is well wrung out, and after the patient is taken out of it and hot bottles are placed about her, be sure that none of the bottles be allowed to touch the skin, or a bad burn will result. The vitality of the tissues is so lowered, that a bottle which would not burn an ordinary patient will cause a serious burn to an eclamptic. Hot poultices are often applied over the loins and the back of the chest, and these, if very hot, may also cause serious burning. In a case recently admitted to the Maternity Hospital the whole of the back was burned, in some places to the third degree.

During the actual struggles, chloroform is given to control the fits. Many employ morphia, but I have entirely given up using it. If the patient is very restless, I usually give her a large dose of chloral hydrate.

At one time, bleeding was freely resorted to, but it went out of fashion for this, as for other diseases. There is no doubt, however, that it is a most useful method of treatment. In most of my recent cases I have used it, with extremely good results. A vein in the arm is opened, and from twelve to twenty ounces of blood are abstracted, and then the saline solution is run directly into the vein. A certain amount of the poison is removed in the blood withdrawn, and the remainder of the poison is well diluted by the saline solution injected.

In cases not actually in labour, the obstetrical part of the treatment is difficult to decide upon. If the fits do not yield to treatment, the uterus should be emptied, but if the fits cease, there is no occasion for such heroic treatment. When labour is going on, delivery should be hastened, and free bleeding should be encouraged in the third stage. The fits often cease after delivery, but this is not always the case, and, as you know, they sometimes begin during the puerperium. In such cases, the treatment should be the same as in those occurring before delivery.

The after-treatment of the patient is the same as for an ordinary puerperal case, only the bowels must be kept very free, and the patient be strictly confined to milk diet, until the albumen has disappeared from the urine. Stimulants are often necessary, and great care must be taken to guard against chills, as lung complications are apt to occur.

